# Mechanisms of linezolid resistance in *Staphylococcus capitis* with the novel mutation C2128T in the 23S rRNA gene in China

**DOI:** 10.1186/s12866-022-02616-9

**Published:** 2022-08-20

**Authors:** Xiao Han, Guiling Zou, Jiaren Liu, Chun Yang, Xuefei Du, Guoyu Chen, Zhe Sun, Xinyu Zhang, Yu Sun, Wanying Zhang, Xiaofeng Jiang

**Affiliations:** 1 The Department of Clinical Laboratory, The Fourth Affiliated Hospital of Harbin Medical University, 37 Yiyuan Street, Nangang District, Harbin, 150001 China; 2 Heilongjiang Longwei Precision Medical Laboratory Center, Harbin, China

**Keywords:** Linezolid resistance, C2128T mutation, Coagulase-negative Staphylococcus, Chloramphenicol-florfenicol resistance, 23S rRNA gene

## Abstract

**Purpose:**

The objective of this study was to investigate the molecular characteristics and potential resistance mechanisms of linezolid-resistant (LZR) *Staphylococcus capitis* isolates from a tertiary hospital in China.

**Methods:**

*S. capitis* isolates were obtained from clinical patient specimens; three of the isolates came from blood cultures and one from the hydrothorax. The agar dilution and E-test methods were used to identify antibiotic resistance. The chloramphenicol-florfenicol resistance (*cfr*) gene carrier status of the strains was determined by PCR. Whole-genome sequencing (WGS) was used to identify point mutations and L3, L4, and L22 mutations and to study the genetic environment of the *cfr* gene and the relationships between strains.

**Results:**

The 4 isolates obtained in this study were all linezolid-resistant *Staphylococcus* strains. A similar of susceptibility profile pattern was observed in all four *S. capitis* strains, each of which exhibited a multidrug-resistant phenotype. A potentially novel mutation, C2128T, was identified, and the *cfr* genes of *S. capitis* strains were all positive. Additionally, the same mutations (C2128T and G2600T) were identified in all 23S rRNA sequences of the isolates, whereas mutations were lacking in the L3, L4, and L22 ribosomal proteins. The genetic environments surrounding *cfr* were identical in all four isolates. A schematic diagram of the phylogenetic tree showed that they were closely related to AYP1020, CR01, and TW2795, and a total of seven drug resistance genes were identified in these strains.

**Conclusions:**

The study indicated that the resistance of the *Staphylococcus capitis* strains to linezolid was caused by multiple mechanisms, and a potential novel mutation, C2128T, that may have an impact on bacterial resistance was identified.

**Supplementary Information:**

The online version contains supplementary material available at 10.1186/s12866-022-02616-9.

## Introduction

*Staphylococcus capitis*, a coagulase-negative staphylococcus (CoNS) bacterium, is a normal component of the human skin tissue microbiota that can cause diseases of the body under certain conditions, including skin infections, bloodstream infections, and even sepsis [1, 2]. In recent years, due to the wide application of a wide range of broad-spectrum antibacterial drugs, immunosuppressants, and chemotherapy drugs in the clinic and the widespread implementation of various invasive surgeries, the bacterial harvest rate has increased each year, and drug resistance has continuously risen. At present, the phenomenon of bacterial drug resistance is increasing at an alarming rate [3]. Linezolid was the first oral oxazolidinone antibiotic approved by the U.S. Food and Drug Administration (FDA) for clinical applications in 2000 [4]. This antibiotic is active against a broad spectrum of gram-positive pathogens, including methicillin-resistant *Staphylococcus aureus* (MRSA), *Streptococcus pneumoniae*, and vancomycin-resistant *Enterococci* (VRE) [5]. Linezolid inhibits bacterial protein production by binding to rRNA on the 50S ribosomal subunit [6]. Linezolid is primarily used to block the development of the initiation complex during protein synthesis; it can inhibit the formation of the initiation complex but does not affect the elongation or termination of the peptide chain during protein synthesis [7].

The incidence of linezolid resistance is gradually increasing with the widespread use of linezolid. There have been many previous reports of linezolid-resistant (LZR) *S. capitis* infections. According to previous studies, the best-defined mechanism of change in LZR is a mutation of the 23S rRNA gene target and acquisition of the chloramphenicol-florfenicol resistance (*cfr*) gene [8–10]. 23S rRNA is a component of the bacterial ribosome 50S subunit, and 23S rRNA mutations can arise during exposure to linezolid; hence, the drug stops working, eventually leading to drug resistance. Gene mutation sites also vary among different strains [11, 12]. G2447U and G2576U gene mutations are the most common in *Staphylococcus* [10, 13]. *Cfr*, which was first discovered in plasmids of *Staphylococcus* isolates from German cows, encodes a methyltransferase that can modify 23S rRNA [14]. The *cfr* gene can mediate the methylation at large subunit sites of ribosomes, causing linezolid resistance [15, 16].

In addition, LZR *S. aureus* was reported in the first year of the clinical use of linezolid in China [17]. Since then, LZR *Staphylococcus* isolates have been successively documented in the United States [18], Mexico [19], Japan [20], Spain [21], and Italy [22]. Therefore, there is an urgent need to elucidate the resistance mechanism of LZR in China. This study was conducted to investigate the clinical features of LZR *S. capitis* found in clinical infections, to further explore its molecular characteristics and mechanisms of resistance and provide a clinical treatment for the prevention of LZR *S. capitis*.

## Materials and methods

### Bacterial isolates and patients

From 2018 to 2019, the study of 4 linezolid-resistant bacterial strains isolated from patients and associated data was authorized by the ethics committee of the Fourth Affiliated Hospital of Harbin Medical University, China. Before their inclusion in the current study, all patients signed a written informed consent form. Four *Staphylococcus capitis* isolates resistant to linezolid were identified in the study, all of which were included in further analyses. The strains were first isolated and identified using the VITEK 2 COMPACT system (VITEK 2 Compact, Biomerieux, France) and then confirmed by 16S sequencing. The isolates were recovered from patients with chronic obstructive pulmonary disease (COPD), acute coronary syndrome, rectal cancer, and a male pelvic abscess. Clinical data of the patients harbouring each isolate, including their age, sex, prior exposure to linezolid, and clinical outcome, were obtained retrospectively. The data were kept anonymous.

### Antimicrobial susceptibility testing

The tested antimicrobial agents include oxacillin, penicillin, gentamicin, ciprofloxacin, levofloxacin, moxifloxacin, erythromycin, clindamycin, quinupristin/dalfopristin, vancomycin, tetracycline, tigecycline, rifampicin, trimethoprim/sulfamethoxazole, and linezolid, as recommended by the Clinical and Laboratory Standards Institute (CLSI) guidelines [23]. The minimal inhibitory concentration (MIC) (VITEK 2 Compact, Biomerieux, France) of every antibiotic was calculated via the agar dilution MIC method, and the results were interpreted according to the CLSI. The MIC of linezolid was also calculated with the E-test (Biomerieux, France) according to the instructions provided by the manufacturer. *S. aureus* ATCC 25923 and ATCC 29213 were tested concurrently for quality control (both showed a linezolid MIC of ≤1 μg/ml, Laboratory Department of the Fourth Affiliated Hospital of Harbin Medical University, China).

### PCR identification of *cfr*

PCR was used to determine the existence of *cfr* in *S. capitis*. The colonies harvested from agar plates were incubated for 5 minutes in 500 μl of H_2_O at a temperature of 100 °C. Following 2 minutes of centrifugation, 1 μl of the resultant supernatant was used as a template for PCR conducted using two *cfr*-specific primers (forward primer: 5*′*-GAAGCTCTAGCCAACCGTCA-3*′*, reverse primer: 5*′*-TCTACCTGCCCTTCGTTTGC-3*′*, amplicon size: 458 base pairs (bp), overall gene fragment size: 1050 bp, GenBank reference sequence: AM408573). The amplification conditions were as follows: 5 minutes at a temperature of 94 °C; 30 cycles of 94 °C for 30 seconds, 55 °C for 30 seconds, and 72 °C for 1 minute; and a final extension step of 72 °C for 7 minutes. The PCR products were imaged by using a visible light transilluminator (Bioteke, Beijing, China).

### Whole-genome sequencing (WGS)

Products of bacterial genomic DNA were sequenced on the Illumina HiSeq (Illumina, America) and PacBio RS (Pacific Biosciences, America) platforms after they were extracted and purified by using a purification kit (TaKaRa, Dalian, China). On the basis of the 16S rRNA nucleotide sequence, a phylogenetic analysis was performed. The obtained 16S and 23S genome sequences are stored in GenBank (accession numbers SUB11152030 and SUB11152217, respectively). The four strains’ representative 16S rRNA nucleotide sequences were compared against the sequences of other *Staphylococcus* strains stored in GenBank (Supplementary materials). The evolutionary history was inferred using the neighbour-joining method [24]. The optimal tree with a sum of branch length = 0.10605004 is shown. The percentage of replicate trees in which the associated taxa clustered together in the bootstrap test (500 replicates) is shown next to the branches [25]. The tree was drawn to scale with branch lengths in the same units as the evolutionary distances used to infer the phylogenetic tree. The evolutionary distances were computed using the p-distance method and are presented in units of the number of base differences per site. The analysis involved 27 nucleotide sequences. The included codon positions were 1st + 2nd + 3rd + Noncoding. All positions with less than 50% site coverage were eliminated. That was, fewer than 50% of alignment gaps, missing data, and ambiguous bases were allowed at any position. There were a total of 1496 positions in the final dataset. Phylogenetic inferences were made using the neighbour-joining method within MEGA software (version 7.0.26) [26].

### Genetic environment of the *cfr* gene

The genetic context of the *cfr* gene was determined by WGS. The reference sequences of *S. capitis* pXWZ (GenBank reference sequence MT096435), *S. aureus* pSR01 (GenBank reference sequence CP048644), *S. capitis* pcfr-XZ03 (GenBank reference sequence CP077712), and *S. xylosus* pSX01 (GenBank reference sequence KP890694) were downloaded from the National Center of Biotechnology Information (NCBI). Resistance genes were obtained from the Comprehensive Antibiotic Research Database (CARD) (https://card.mcmaster.ca/). The polished assembly report and annotation information of the obtained genomic sequences of the 4 strains are shown in supplementary tables (Supplementary Tables 1 and 2).

## Results

### Characteristics of linezolid-resistant *S. capitis*

The 4 isolates obtained in this study were all linezolid-resistant *Staphylococcus* strains. Three strains of linezolid-resistant *S. capitis* were recovered from blood samples, and one strain was recovered from a pleural effusion sample. Table 1 summarizes the demographic and clinical characteristics of the patients. All patients had received linezolid for an average of 11.5 days leading up to the isolation of linezolid-resistant *S. capitis.* A similar susceptibility profile pattern was observed in the four *S. capitis* strains. All of the isolates exhibited a multidrug-resistant phenotype (resistant to three or more different antibiotics). All isolates were resistant to linezolid (MIC 256 mg/L), penicillin, oxacillin, ciprofloxacin, gentamicin, erythromycin, moxifloxacin, and levofloxacin. Their resistance to clindamycin and quinupristin/dalfopristin was variable (Table 2).

**Table 1:** Clinical information of patients with linezolid-resistant *Staphylococcus capitis* isolates

Isolate	Age (years)/Gender	Underlying diseases	Date of isolation (dd/mm/yy)	Source	Department	Prior linezolid therapy	Time to isolation of LZR ***S. capitis*** (days)	Outcome
**701**	68/M	COPD	04/08/2018	Blood	ICU	Yes	13	Survived
**703**	53/M	Acute coronary syndrome	03/09/2018	Blood	Cardiology	Yes	12	Died
**708**	52/M	Rectal cancer	11/10/2018	Blood	ICU	Yes	8	Survived
**709**	60/M	Male pelvic abscess	10/07/2019	Hydrothorax	ICU	Yes	13	Survived

*ICU* Intensive care unit, *M* Male, *COPD* Chronic obstructive pulmonary disease, *LZR S. capitis* Linezolid-resistant *Staphylococcus capitis*

**Table 2:** Antibiotic susceptibilities and linezolid resistance mechanisms of linezolid-resistant *Staphylococcus capitis* isolates

Isolate	MIC (mg/L)															Genetic resistance markers				
	LZD	P	OXA	CN	CIP	LEV	MXF	E	DA	QD	VA	TE	TGC	RA	SXT	***cfr***	23S rRNA	L3	L4	L22
**701**	≥256	≥0.5	≥4	≥16	≥8	4	2	≥8	2	1	≤0.5	1	0.25	≤0.5	20	+	C2128, G2600T	–	–	–
**703**	≥256	≥0.5	≥4	≥16	≥8	≥8	2	≥8	≥8	2	≤0.5	2	0.5	≤0.5	20	+	C2128T, G2600T	–	–	–
**708**	≥256	≥0.5	≥4	≥16	≥8	4	2	≥8	≥8	2	≤0.5	2	0.5	≤0.5	20	+	C2128T, G2600T	–	–	–
**709**	≥256	≥0.5	≥4	≥16	≥8	4	2	≥8	≥8	2	≤0.5	2	0.5	≤0.5	20	+	C2128T, G2600T	–	–	–

*MIC* Minimum inhibitory concentration, *LZD* Linezolid, *P* Penicillin, *OXA* Oxacillin, *CN* Gentamicin, *CIP* Ciprofloxacin, *LEV* levofloxacin, *MXF* Moxifloxacin, *E* Erythromycin, *DA* Clindamycin, *QD* Quinupristin/dalfopristin, *VA* Vancomycin, *TE* Tetracycline, *TGC* Tigecycline, *RA* Rifampicin, *SXT* Trimethoprim/sulfamethoxazole (19 mg/L /1 mg/L)

### Resistance genes and mutations

The PCR screening results were positive for the *cfr* gene in all isolates, which was further confirmed by sequencing. Additionally, an investigation of the 23S rRNA sequences revealed identical mutations (C2128T and G2600T) in all isolates but no alterations in ribosomal proteins L3, L4, and L22. A potentially novel mutation, C2128T, was identified in all four linezolid-resistant *S. capitis* isolates (GenBank accession number SUB11152217).

### Genetic environment of the *cfr* gene in the plasmids

**Fig. 1:**
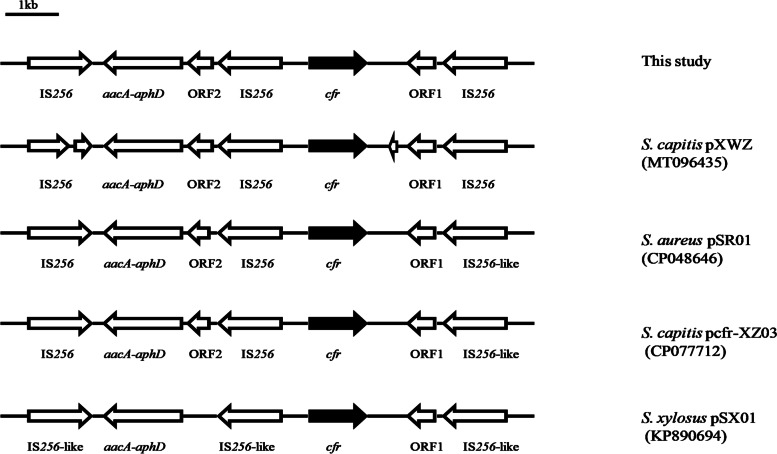
Schematic diagram of the genetic environment of the *cfr* gene in this study. The arrows represent the positions and direction of the elements. The *cfr* gene is shown in black

The genetic environment surrounding *cfr* was similar in all four isolates (Fig. 1). A complete Tn*4001*-like transposon was identified upstream of *cfr* in the plasmid. The aminoglycoside resistance gene pair *aacA-aphD* carried by Tn*4001* was flanked by two IS*256* elements containing transposase genes. Another copy of the IS*256*-associated transposase gene was discovered downstream of *cfr*; this copy was oriented towards the *cfr* gene and identical in sequence to the right (*cfr*-proximal) transposase gene of the Tn*4001*-like transposon. This transposase gene was followed by a short open reading frame 1 (ORF1) encoding a protein with similarity to a transcriptional regulator of a helix-turn-helix (HTH) protein. The genetic environment surrounding the *cfr* gene exhibited striking similarity to those of previously reported plasmids, including *pcfr*-XZ03 (100% coverage, 100% identity, CP077712) and pXWZ (100% coverage, 100% identity, MT096435) from *S. capitis* strains, pSR01 (100% coverage, 99.98% identity, CP048646) from *S. aureus* strains, and pSX01 (99% coverage, 99.97% identity, KP890694) from an *S. xylosus* strain. These findings are consistent with the spread of *cfr*-positive sequences among different species and genera via various health care facilities.

### Genetic relationship with other strains and drug resistance genes

**Fig. 2:**
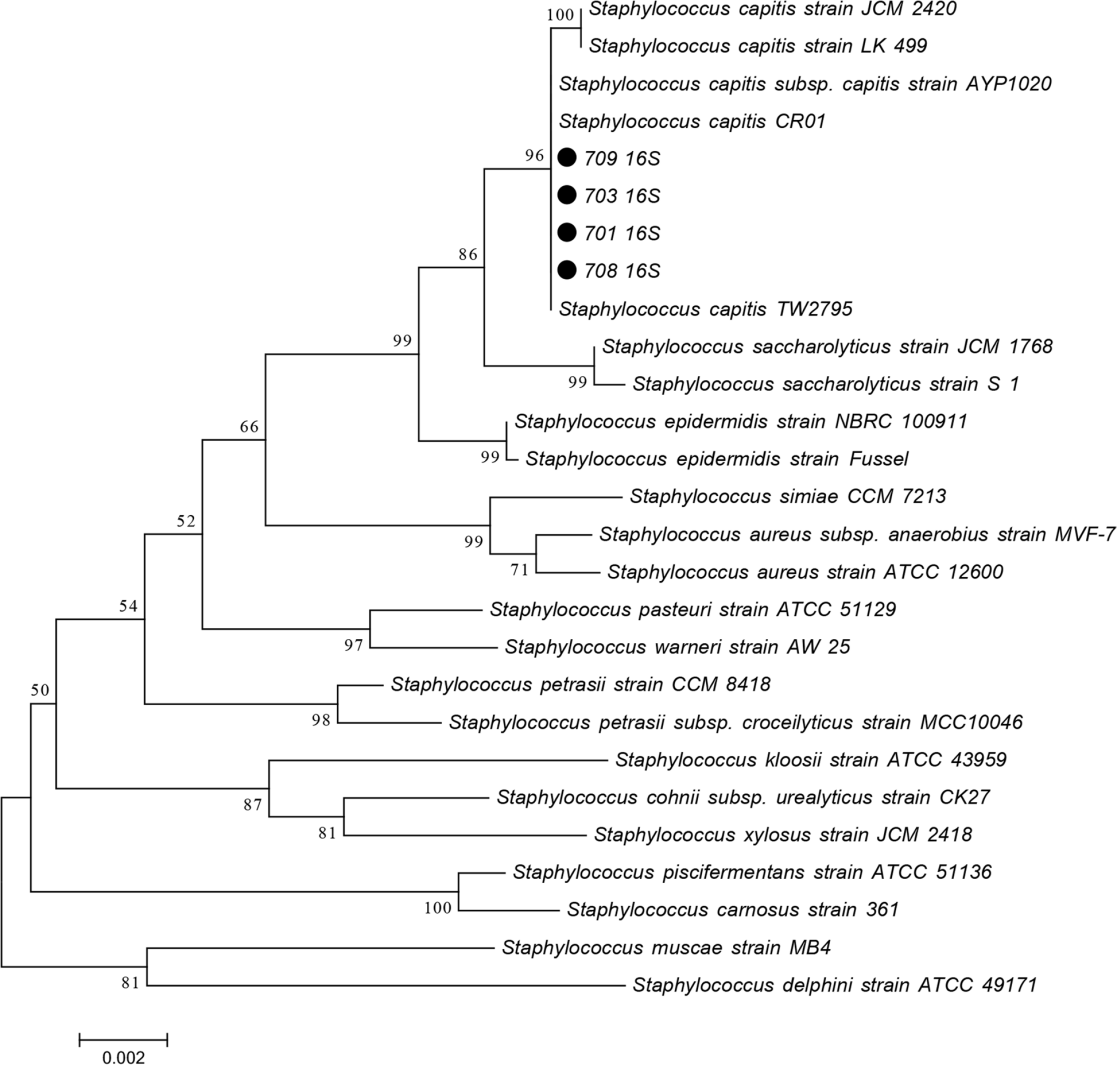
Schematic diagram of the phylogenetic tree of the four strains. The strains of the research group have been marked with emphasis. The strains and their corresponding GenBank accession numbers for 16S rRNA genes are shown following the organism names. Numbers at the branching nodes are percentages of bootstrap values based on 500 replications. Bootstrap values greater than 50% are shown at the branch points. The scale bar represents 0.002 substitutions per nucleotide position

The 16S rRNA genes of the four linezolid-resistant strains were identical to the sequences of the corresponding region (GenBank accession number SUB11152030), and they were more closely related to AYP1020, CR01, and TW2795 than to other strains (Fig. 2). The comparison with the CARD revealed that the resistance genes carried by the four linezolid-resistant strains of *S. capitis* were identical to those shown in Table 3. There were a total of seven drug resistance genes, among which *cfr, blaZ, qacA,* and *aac(6′)-aph(2″)* were located on the plasmids.

**Table 3:** Drug resistance genes from WGS

Isolates	Aminoglycoside	Beta-lactam	Macrolide	Oxazolidinone	Phenicol	Disinfectant
**701**	*AadD;aac(6′)-aph(2″); aadD; ant(9)-Ia*	*mecA;blaZ*	*ermA;cfr*	*cfr*	*cfr*	*qacA*
**703**	*AadD;aac(6′)-aph(2″); aadD; ant(9)-Ia*	*mecA;blaZ*	*ermA;cfr*	*cfr*	*cfr*	*qacA*
**708**	*AadD;aac(6′)-aph(2″); aadD; ant(9)-Ia*	*mecA;blaZ*	*ermA;cfr*	*cfr*	*cfr*	*qacA*
**709**	*AadD;aac(6′)-aph(2″); aadD; ant(9)-Ia*	*mecA;blaZ*	*ermA;cfr*	*cfr*	*cfr*	*qacA*

*WGS* Whole-genome sequencing

## Discussion

Health care settings have seen a notable increase in the isolation of drug-resistant strains since the extensive use of linezolid was initiated. In the current study, we determined the incidence of *the cfr* gene and mutations of C2128T and G2600T in 23S rRNA that existed in linezolid-resistant *S. capitis* isolates recovered from 4 patients from a tertiary hospital in China. In general, *cfr* methyltransferase mediates resistance to antibiotics targeting the 50S ribosome subunit, including florfenicol, lincosamide, oxazolidinone, pleuromutilin, and streptomycin [10]. Thus, *cfr*-carrying *staphylococci* exhibit a multidrug-resistant phenotype that is consistent with the resistance profile of the isolates obtained in this study. A novel mutation was identified in the V region of the 23S rRNA, located near the linezolid binding site. Notably, the *cfr* gene was shown to be carried by plasmids, making it amenable to horizontal transmission across *staphylococci*. Previously, it was shown that the *cfr gene* plays a critical role in mediating linezolid resistance. The isolates expressing the *cfr gene* exhibited significantly higher levels of linezolid resistance than the isolates without the *cfr gene* [9]. The observation of a high prevalence of *cfr* in our drug-resistant strains raises the possibility of horizontal gene transfer, which is a reminder of the need for a proactive approach to infection control.

In addition, previous studies have reported that His 146 and Gly155Arg in L3 from a research lab strain of *S. aureus* and a mutation in the conserved region (63KPWRQKGTGRAR74) of the L4 protein are all related to the cross-resistance of *S. pneumoniae*, *S. aureus*, and *C. perfringens* to linezolid [27–29]. However, the L3, L4, and L22 ribosomal proteins of the four linezolid-resistant isolates studied in our investigation did not exhibit similar mutations. Furthermore, a unique mutation, C2128T, was identified in the 23S rRNA gene; this mutation was distinct from the typical changes in the central loop of 23S rRNA domain V observed in most linezolid-resistant strains, such as *Enterococcus* and *Staphylococcus*. To the best of our knowledge, resistance to linezolid has been attributed to G2528U, G2576U, and G2505A (*Enterococcus* strains); G2447U, G2576U and G2603T (*Staphylococcus* strains); and C2534T, G2447T, G2576T, T2504A, C2109T and G2474T (*Coagulase-negative Staphylococci* strains) [13, 30, 31]. Notably, there was an absence of the most prevalent mutation, G2576T, in the V region of the 23S rRNA gene in all four linezolid-resistant isolates, whereas the G2600T mutation was identified in all isolates. Additionally, investigations have discovered a correlation between the prevalence of linezolid resistance and the number of mutations in a copy of the 23S rRNA-encoding gene [32]. The close genetic relatedness of the clones examined in this study to the CR01 and AYP1020 genomes was surprising, as both CR01 and AYP1020are fully susceptible strains. To date, no explanation has been suggested for this unexpected clustering. Both lineages may represent preresistance lineages of linezolid clones, but there is presently no epidemiological evidence to substantiate this hypothesis [33].

Since 3 of the strains of LZR *S. capitis* included in this study were recovered from blood samples, it is important for clinicians to perform blood cultures to confirm the presence of bloodstream infections. The timely detection of linezolid-resistant *staphylococci* in the early stage is of great significance for ensuring optimal antibiotic treatment and limiting the emergence of multidrug-resistant bacteria. A previous report showed that the mean isolation time of *linezolid-resistant coagulase-negative staphylococci* (LRCoNS) strains was 11.0 ± 8.0 days in patients following linezolid treatment, but resistant strains were obtained in a few cases because of cross-infection [13]. This may provide more substantial proof of the theory that the use of linezolid is a potential independent risk factor for the development of LRCoNS strains [34]. This has been shown by the finding that all patients carrying linezolid-resistant *S. capitis* had taken linezolid medication for an average of 11.5 days prior to the harvesting of the linezolid-resistant *S. capitis* in our investigation. Taking into consideration that the same drug-resistant clones were recovered from these patients and the time periods during which the strains were harvested, we hypothesize that this could have been due to clonal propagation between strains 701, 703, and 708 [35]. While the incidence of LZR *staphylococci* remains low, factors such as prolonged hospitalization, numerous interventions, and irrational antibiotic use may hasten the emergence and spread of LZR *staphylococci*. The discreet use of linezolid and monitoring of staphylococcal resistance are necessary for therapeutic efficacy.

## Conclusion

In conclusion, we analysed mutations in the 23S rRNA of 4 LZR *S. capitis* strains, together with isolated quality control strains and sensitive strains such as AYP1020 and CR01, to identify the potentially novel mutation C2128T, which may have an impact on linezolid resistance. This work provides new ideas for improving the rational use of antibacterial drugs and the prevention and control of hospital infections in the future and minimizing the emergence of multidrug resistance.

## Supplementary Information

**Additional file 1.**

**Additional file 2: Supplementary Table 1.** Polished Assembly Report of 4 strains. **Supplementary Table 2.** Annotation Information of 4 strains.

## Data Availability

The datasets generated and/or analyzed during the current study are available in the GenBank repository, https://cipotato.org/genebankcip/.
