# Supplementary material for: Mechanisms of linezolid resistance in Staphylococcus capitis with the novel mutation C2128T in the 23S rRNA gene in China
Source: BMC Microbiol. 2022 Aug 20;22:203. doi: 10.1186/s12866-022-02616-9 (PMC9392311; doi:10.1186/s12866-022-02616-9)
Supplement: Supplementary file 1 — Additional file 1. [file 12866_2022_2616_MOESM1_ESM.docx]

The following reference genomes were used for comparisons: Staphylococcus capitis JCM 2420 (GenBank reference sequence NR_113348.1), Staphylococcus capitis LK 499 (GenBank reference sequence NR_036775.1), Staphylococcus capitis subsp. Capitis strain AYP1020 (GenBank reference sequence NZ_CP007601.1), Staphylococcus delphini strain ATCC 49171 (GenBank reference sequence NR_024666.1), Staphylococcus kloosii strain ATCC 43959 (GenBank reference sequence NR_024667.1), Staphylococcus pasteuri strain ATCC 51129 (GenBank reference sequence NR_024669.1), Staphylococcus warneri strain AW 25 (GenBank reference sequence NR_025922.1), Staphylococcus carnosus strain 361 (GenBank reference sequence NR_027518.1), Staphylococcus saccharolyticus strain S 1 (GenBank reference sequence NR_029158.1), Staphylococcus aureus subsp. anaerobius strain MVF-7 (GenBank reference sequence NR_036828.1), Staphylococcus epidermidis strain Fussel (GenBank reference sequence NR_036904.1), Staphylococcus cohnii subsp. urealyticus strain CK27 (GenBank reference sequence NR_037046.1), Staphylococcus simiae CCM 7213 (GenBank reference sequence NR_043146.1), Staphylococcus muscae strain MB4 (GenBank reference sequence NR_104762.1), Staphylococcus piscifermentans strain ATCC 51136 (GenBank reference sequence NR_112035.1), Staphylococcus xylosus strain JCM 2418 (GenBank reference sequence NR_113350.1), Staphylococcus saccharolyticus strain JCM 1768 (GenBank reference sequence NR_113405.1), Staphylococcus epidermidis strain NBRC 100911 (GenBank reference sequence NR_113957.1), Staphylococcus petrasii strain CCM 8418 (GenBank reference sequence NR_118450.1), Staphylococcus aureus strain ATCC 12600 (GenBank reference sequence NR_118997.2), Staphylococcus petrasii subsp. croceilyticus strain MCC10046 (GenBank reference sequence NR_132590.1), (GenBank reference sequence ), Staphylococcus capitis CR01 (GenBank reference sequence GCF_000499705.1), Staphylococcus capitis TW2795 (GenBank reference sequence AP014956.1).
