# Supplementary material for: Mechanisms of linezolid resistance in Staphylococcus capitis with the novel mutation C2128T in the 23S rRNA gene in China
Source: BMC Microbiol. 2022 Aug 20;22:203. doi: 10.1186/s12866-022-02616-9 (PMC9392311; doi:10.1186/s12866-022-02616-9)
Supplement: Supplementary file 2 — Additional file 2: Supplementary Table 1. Polished Assembly Report of 4 strains. Supplementary Table 2. Annotation Information of 4 strains. [file 12866_2022_2616_MOESM2_ESM.docx]

**Supplementary Table 1.** Polished Assembly Report of 4 strains

| Analysis Metric | 701 | 703 | 708 | 709 |
| --- | --- | --- | --- | --- |
|  |  |  |  |  |
| Polished Contigs | 5 | 4 | 3 | 4 |
| Maximum Contig Length | 2569712 | 2569712 | 2569712 | 2569719 |
| N50 Contig Length | 2569712 | 2569712 | 2569712 | 2569719 |
| Sum of Contig Lengths | 2850531 | 2840130 | 2692935 | 2763220 |
| E-size (sum of squares / sum) | 2325513.82 | 2335276.53 | 2455171.22 | 2394719.03 |

**Supplementary Table 2.** Annotation Information of 4 strains

| Metric | 701 | 703 | 708 | 709 |
| --- | --- | --- | --- | --- |
| Genome Size | 2569712 bp | 2569712 bp | 2569712 bp | 2569712 bp |
| G+C content | 32.9% | 32.9% | 32.9% | 32.9% |
| Is circular | Y | Y | Y | Y |
| Number of Plasmid | 3 | 3 | 3 | 3 |
| Number of genes | 2446 | 2446 | 2447 | 2447 |
| Avg.gene length | 905.1 bp | 905.1 bp | 904.7bp | 904.7bp |
| CDS region ratio | 86.20% | 86.20% | 86.10% | 86.20% |
